# Fluid shear stress-induced TGF-β/ALK5 signaling in renal epithelial cells is modulated by MEK1/2

**DOI:** 10.1007/s00018-017-2460-x

**Published:** 2017-02-06

**Authors:** Steven J. Kunnen, Wouter N. Leonhard, Cor Semeins, Lukas J. A. C. Hawinkels, Christian Poelma, Peter ten Dijke, Astrid Bakker, Beerend P. Hierck, Dorien J. M. Peters

**Affiliations:** 1 Department of Human Genetics, Leiden University Medical Center, 2300 RC Leiden, The Netherlands; 2 Department of Oral Cell Biology, Academic Centre for Dentistry Amsterdam (ACTA), University of Amsterdam and VU University Amsterdam, 1081 LA Amsterdam, The Netherlands; 3 Department of Molecular Cell Biology, Cancer Genomics Centre Netherlands, Leiden University Medical Center, 2300 RC Leiden, The Netherlands; 4 Department of Gastroenterology-Hepatology, Leiden University Medical Center, 2300 RC Leiden, The Netherlands; 5 Laboratory for Aero and Hydrodynamics, Delft University of Technology, 2628 CA Delft, The Netherlands; 6 Department of Anatomy and Embryology, Leiden University Medical Center, 2300 RC Leiden, The Netherlands

**Keywords:** Fluid flow, Mechanotransduction, Cilia, SMAD2/3 signaling, ERK1/2, *Pkd1*^−/−^

## Abstract

Renal tubular epithelial cells are exposed to mechanical forces due to fluid flow shear stress within the lumen of the nephron. These cells respond by activation of mechano-sensors located at the plasma membrane or the primary cilium, having crucial roles in maintenance of cellular homeostasis and signaling. In this paper, we applied fluid shear stress to study TGF-β signaling in renal epithelial cells with and without expression of the *Pkd1*-gene, encoding a mechano-sensor mutated in polycystic kidney disease. TGF-β signaling modulates cell proliferation, differentiation, apoptosis, and fibrotic deposition, cellular programs that are altered in renal cystic epithelia. SMAD2/3-mediated signaling was activated by fluid flow, both in wild-type and *Pkd1* ^−/−^ cells. This was characterized by phosphorylation and nuclear accumulation of p-SMAD2/3, as well as altered expression of downstream target genes and epithelial-to-mesenchymal transition markers. This response was still present after cilia ablation. An inhibitor of upstream type-I-receptors, ALK4/ALK5/ALK7, as well as TGF-β-neutralizing antibodies effectively blocked SMAD2/3 activity. In contrast, an activin-ligand trap was ineffective, indicating that increased autocrine TGF-β signaling is involved. To study potential involvement of MAPK/ERK signaling, cells were treated with a MEK1/2 inhibitor. Surprisingly, fluid flow-induced expression of most SMAD2/3 targets was further enhanced upon MEK inhibition. We conclude that fluid shear stress induces autocrine TGF-β/ALK5-induced target gene expression in renal epithelial cells, which is partially restrained by MEK1/2-mediated signaling.

**Electronic supplementary material:**

The online version of this article (doi:10.1007/s00018-017-2460-x) contains supplementary material, which is available to authorized users.

## Introduction

Cellular mechano-sensitivity plays fundamental roles in cell viability and function, tissue development, and maintenance of organs [1]. For example, the kidney has the capacity to increase glomerular filtration rate in response to physiological stimuli. In addition, in renal diseases, hyperfiltration usually occurs in the remaining functional nephrons to compensate for the lost glomeruli and nephrons [2]. Fundamental in the regulation of altered fluid shear stress are primary cilia and other mechano-sensors, and defects in cilia formation and function have profound effects on the development of body pattern and the physiology of multiple organ systems [3]. The signaling modules responsible for the flow-sensing response involve a number of proteins located in the cell membrane, cilium and/or at the ciliary base, including polycystin-1 (PC-1) and the ion channel polycystin-2 (PC-2), encoded by the genes mutated in patients with autosomal dominant polycystic kidney disease (ADPKD) [4, 5]. At the plasma membrane and in cilia, polycystins interact with diverse (mechanosensory) ion channels, signal transducers as well as cell–cell and cell–extracellular matrix junctional proteins [6–11]. Therefore, the polycystins are thought to play a role in differentiation and maintenance of the cell structure, mechanical force transmission, and mechanotransduction [1, 12, 13]. Lack of the polycystin complex in cilia is one of the proposed mechanisms of renal cyst formation [14, 15]. Moreover, mutations or deletions of other ciliary proteins can also cause renal cystic disease in mouse models and patients, indicating the role of cilia during cystogenesis [16, 17]. In the absence of polycystins, renal epithelial cells lack the capability to respond to signals needed to maintain the epithelium differentiated, finally resulting in cyst formation [15].

Primary cilia also play essential roles as signal transducers in growth factor signaling. Ligands in the tubular fluid flow bind to their receptors, inducing cellular responses through downstream signaling pathways, for instance affecting the hedgehog (Hh), epidermal growth factor receptor (EGFR), Wnt and transforming growth factor β (TGF-β) pathways [3, 18]. Although not exclusively, receptors involved in these pathways have been identified in the cilium of several cell types, including renal epithelial cells, suggesting that different signaling cascades are being regulated by this organelle [3, 18–20]. The above-mentioned data indicate that primary cilia are essential in organizing different signaling systems that sense environmental cues and transmit signals to the cell interior. Gene expression and the overall cellular behavior will be the effect of an integration of the different signaling pathways, triggered by flow and by growth factor or cytokine stimulation.

A cytokine previously reported to be involved in fluid flow and shear stress-regulated signaling is TGF-β [21, 22]. The TGF-β superfamily members are multifunctional cytokines and include among others TGF-βs, activins, and bone morphogenetic proteins (BMPs). TGF-β signaling modulates cell proliferation, differentiation, apoptosis, adhesion, and cell migration, and is believed to play a crucial role in fibrotic deposition [23], which is seen in cyst formation [24]. TGF-β, as well as activin and Nodal, binds to a pair of serine/threonine kinase transmembrane receptors that mediate the phosphorylation of receptor-regulated SMAD2 and 3. These phosphorylated SMAD proteins, p-SMAD2 and -3, form a complex with SMAD4 and can enter the nucleus where they act as transcription factors to regulate the transcription of various genes.

In embryonic endothelial cells, shear stress-mediated TGF-β/activin receptor-like kinase 5 (ALK5) signaling induced endothelial-to-mesenchymal transition, depending on the strength of shear and presence or absence of a cilia [21, 25]. A similar type of observation was made for renal epithelial cells, where fluid shear stress dynamically regulated TGF-β gene expression and SMAD3 activation, depending on the magnitude of fluid shear, i.e. physiological versus pathological, and depending on NOTCH4 expression [22, 26]. Increased SMAD2/3 activation and increased TGF-β signaling has been shown in several animal models for renal cystic disease and patient-derived tissues [24, 27].

Given the role for SMAD2/3 signaling in shear stress but also in cyst formation, we aim to characterize in this study the cellular response of renal epithelial cells to fluid shear stress by unraveling the signaling cascades, particularly focusing on SMAD2/3 signaling and the effect of MAPK/ERK signaling. Our data indicate that both SMAD2/3 and epithelial-to-mesenchymal transition (EMT) processes are altered upon fluid flow stimulation in proximal tubular epithelial cells (PTEC) with and without *Pkd1*-gene expression, as shown by phosphorylation of SMAD2/3 and nuclear translocation of p-SMAD2/3 and Snail. This leads to altered expression of target genes and EMT markers, shown in ciliated and non-ciliated cells. These processes are regulated by an interplay between SMAD2/3 and ERK1/2 signaling, and can be partially modulated by upstream ALK4/5/7 and MEK1/2 inhibitors and TGF-β neutralizing antibodies, while the soluble activin receptor-IIB fusion protein (sActRIIB-Fc) was ineffective. We conclude that the fluid shear stress response in PTECs is TGF-β/ALK5 dependent and can be modulated by MAPK/ERK signaling.

## Materials and methods

### Antibodies

SMAD2 (L16D3; #3103) and Snail (C15D3; #3879) antibodies were from Cell Signaling Technology. Acetylated α-tubulin (clone 6-11B-1; #T6793) antibody and Phalloidin-Atto 594 (#51927) were from Sigma-Aldrich. Antibody against α-tubulin (DM1A; #CP06) was from Calbiochem, Merck Millipore. Antibodies against p-SMAD2 and p-SMAD3 have been described previously [28, 29]. Goat anti-Rabbit IgG (H + L) Alexa Fluor 488 conjugate (#A-11008), Goat anti-Mouse IgG (H + L) Alexa Fluor 488 conjugate (#A-11029), and Goat anti-Mouse IgG (H + L) Alexa Fluor 594 conjugate (A-11032) were from Life Technologies. Goat-anti-Rabbit IRDye 800CW (#926-32211) and Goat-anti-Mouse IRDye 680 (#926-32220) were from LI-COR Biosciences.

### Chemicals

ALK4/5/7 inhibitor LY-364947 (10 µM; Calbiochem; #616451) was from Merck Millipore and SB431542 (10 µM; #1614) was from Tocris Bioscience. TGF-β-neutralizing antibody (clone 2G7) was a gift from Dr. E. de Heer (Pathology, LUMC, Leiden); sActRIIB-Fc was a gift from Prof. Olli Ritvos (Haartman Institute, Helsinki, Finland). MEK1/2 inhibitor Trametinib (GSK1120212; #S2673) was from Selleckchem. Recombinant human TGF-β1 (#100-21) and recombinant human TGF-β2 (#100-35B) were purchased from PeproTech. Recombinant human/mouse/rat activin A (#338-AC) and recombinant human activin B (#659-AB) were from R&D systems.

### Cell culture

SV40 large T antigen-immortalized murine proximal tubular epithelial cells (PTEC) (*Pkd1* ^wt^ and *Pkd1* ^−/−^), derived from a *Pkd1* ^lox,lox^ mouse, were generated and cultured as described previously [30]. Cells were maintained at 37 °C and 5% CO_2_ in DMEM/F-12 with GlutaMAX (Gibco, Life Technologies; #31331-093) supplemented with 100 U/ml Penicillin–Streptomycin (Gibco, Life Technologies; #15140-122), 2% Ultroser G (Pall Corporation, Pall BioSepra, Cergy St Christophe, France; #15950-017), 1× Insulin–Transferrin–Selenium–Ethanolamine (Gibco, Life Technologies; #51500-056), 25 ng/l Prostaglandin E1 (Sigma–Aldrich; #P7527), and 30 ng/l Hydrocortisone (Sigma–Aldrich; #H0135). Cell culture was monthly tested without mycoplasma contamination using MycoAlert Mycoplasma Detection Kit (Lonza; LT07-318). New ampules were started after 15 passages.

For growth factor stimulation or fluid flow experiments, cells were cultured on collagen-I (Advanced BioMatrix; #5005) coated culture dishes or glass slides. Prior to treatment, cells were serum-starved to exclude effects of serum-derived growth-factors and to synchronize cells and cilia formation. For growth factor stimulation, 100% confluent cells were serum-starved overnight and incubated with the specified ligands in the absence of medium supplements. Stimulation was done with 5 ng/ml TGF-β1 or TGF-β2 or 100 ng/ml activin A or activin B, unless differently specified. For fluid flow stimulation, cells grown until high confluency underwent 24 h serum starvation before the start of the treatment. Cilia formation was checked on a parallel slide by immunofluorescence using anti-acetylated α-tubulin antibodies (Sigma Aldrich; #T6793). ALK4/5/7 inhibitor (10 µM), MEK1/2 inhibitor (10 µM) or DMSO control (0.1%) were added 1 h before start of ligand or flow stimulation in the absence of medium supplements. To sequester TGF-β or activin ligands, TGF-β neutralizing antibodies (10 µg/ml) or sActRIIB-Fc (5 µg/ml) was added at the start of treatment, by replacing serum-free medium.

### Fluid flow stimulation

Cells were exposed to laminar fluid flow (0.25–2.0 dyn/cm^2^) in a cone–plate device or parallel plate flow chamber. The cone–plate device, adapted from Malek et al. [31, 32], was designed for 3.5 cm cell culture dishes (Greiner Bio-One). Cells were grown on collagen-I-coated dishes until confluence, followed by 24 h serum starvation, before dishes were placed in the cone–plate flow system and incubated at 37 °C and 5% CO_2_. The confluent cell monolayer of 9.6 cm^2^ was subjected to fluid shear stress using 2 ml serum-free DMEM/F-12 medium with viscosity (*μ*) of 0.0078 dyn s/cm^2^ [33]. Constant laminar (*Re* = 0.3) fluid flow was induced using a cone angle (*α*) of 2° and a velocity (*ω*) of 80 rpm, generating a fluid shear stress (*τ* = *μω*/*α*) of 1.9 dyn/cm^2^.

The parallel plate flow chamber was previously described [34, 35]. Briefly, cells were grown on collagen-I-coated glass slides of 36 × 76 mm (Fisher Scientific #15178219) until confluence, followed by 24 h serum starvation, before glass slides were placed in a flow chamber. A confluent cell monolayer of 14.2 cm^2^ (24 × 59 mm) was subjected to fluid shear stress using 7.5 ml serum-free DMEM/F-12 medium. Fluid was pumped at a constant flow rate (Q) of 5.5 ml/min through the chamber with 300 µm height (h), generating a constant laminar (*Re* = 5.0) fluid shear stress (*τ* = 6μQ/h^2^b) of 2.0 dyn/cm^2^, unless differently specified. The parallel plate flow chamber was placed in an incubator at 37 °C and 5% CO_2_.

Static control cells were incubated for the same time in equal amounts of serum-free DMEM/F12 medium at 37 °C and 5% CO_2_. After 4 until 20 h fluid flow or control (static) stimulation, medium was collected and cells have been harvested for mRNA isolation and/or protein isolation for gene expression analysis or western blot. Ammonium sulfate (AS) was used to remove primary cilia as previously described [36]. Cells were pre-treated with 50 mM ammonium sulfate, followed by 6 h fluid flow in serum-free medium or 16 h fluid flow in medium containing 25 mM AS, to prevent cilia restoration. Control cells were treated similarly, but without AS.

### Reporter assay

PTECs were cultured in 3.5 cm culture dishes and transfected after 24 h with 4 µg SMAD3-SMAD4 transcriptional reporter (CAGA_12_-Luc) [37] and 200 ng renilla luciferase reporter as a transfection control (pGL4.75[hRlucCMV]; Promega; #E6931) using 10 µl Lipofectamine 2000 according to the manufacturer’s protocol (Life Technologies; #11668019). Cells were maintained under serum-free conditions from the moment of transfection and fluid flow was started 24 h after transfection. Cells were lysed after 20 h of fluid flow stimulation using a cone–plate device. Firefly and renilla luciferase activities were measured on a luminometer (Victor 3; PerkinElmer) using the Dual-Luciferase Reporter Assay System (#E1960) from Promega according to the manufacturer’s instructions. Firefly luminescence was corrected for renilla to get the relative activity of the reporter.

### Gene expression analysis

Total RNA was isolated from cultured cells using TRI Reagent (Sigma–Aldrich; #T9424) according to manufacturer’s protocol. Gene expression analysis was performed by quantitative PCR (qPCR) as described previously [38]. Briefly, cDNA synthesis was done using Transcriptor First Strand cDNA Synthesis Kit (Roche Applied Science; #04897030001) according to the manufacturer’s protocol. Quantitative PCR was done in triplicate on the LightCycler 480 II (Roche) using 2× FastStart SYBR-Green Master (Roche; #04913914001) according to the manufacturer’s protocol. Data was analyzed with LightCycler 480 Software, Version 1.5 (Roche). Gene expression was calculated using the 2^− ΔΔCt^ method as described previously [39] and normalized to the housekeeping gene *Hprt*, giving the relative gene expression. Mean gene expression and standard deviation of the different treatment groups were calculated. For primer sequences see Supplementary Material 1, Table S1.

### ELISA

Total and endogenously active levels of TGF-β1, TGF-β2, and TGF-β3 in medium collected after fluid flow experiments were determined by ELISA as previously described [40, 41] using ELISA Duosets of TGF-β1 (DY1679), TGF-β2 (DY302), and TGF-β3 (DY243) from R&D systems.

### Western blot analysis

Cells were either scraped in DPBS and 1:1 diluted in 2× RIPA buffer or directly lysed in 1× RIPA buffer (50 mM Tris–HCl, pH 7.4, 150 mM NaCl, 1 mM EDTA, 1% Na-DOC, 1% NP-40). Throughout the lysis procedure, 50 mM NaF, 1 mM Na_2_VO_4_, and 1× complete protease inhibitor cocktail (Roche; #05892970001) were used to inhibit phosphatase and protease activity. Cell lysate was homogenized by three 5 s pulses of sonification followed by 30 min gentle shaking at 4 °C. Insoluble cell debris was removed by 15 min centrifugation at 14,000×*g*. Protein concentration was determined using Pierce BCA protein assay kit (ThermoFisher Scientific; #23227).

Western blot was performed on total protein cell extracts using p-SMAD2, SMAD2, p-SMAD3 or tubulin antibodies. Cell lysates (10–20 µl) were separated on a 10% SDS–PAGE gel. Proteins were transferred to 0.2 µm nitrocellulose membranes (Bio-Rad; #1704158) using Trans-Blot Turbo Transfer System (Bio-Rad; #1704155) at 1.3 A and 25 V for 10 min. Membranes were blocked for 1 h at room temperature in 25% SEA block blocking buffer (ThermoFisher Scientific; #37527) in TBS and incubated overnight at 4 °C with antibodies against p-SMAD2 (1:1000), SMAD2 (1:1000) or p-SMAD3 (1:1000) in 5% bovine serum albumin (BSA) in Tris-buffered saline containing 0.1% Tween-20 (TBST). Tubulin or GAPDH were used as loading controls, with 1 h antibody incubation at room temperature. Goat-anti-Rabbit IRDye 800CW (1:10,000) was used as secondary antibody for the detection of p-SMAD2. Goat-anti-Mouse IRDye 680 (1:12,000) was used as secondary antibody for the detection of total SMAD2 and Tubulin. Horseradish peroxidase conjugated secondary antibody (GE Healthcare, Waukesha, WI, USA) was used for the detection of p-SMAD3 or GAPDH using chemoluminescence according to the manufacturer’s protocol (Pierce, Rockford, IL, USA) as described previously [29]. Detection and densitometric analysis were carried out using the Odyssey Infrared Imaging System (LI-COR-Biosciences). Protein levels were quantified using p-SMAD2/SMAD2-integrated intensity ratios. Tubulin or GAPDH were used as a loading control.

### Immunofluorescence

Cells were fixed in 4% paraformaldehyde and permeabilized in 0.2% Triton-X100 in PBS for 15 min at room temperature. Cells were blocked in 5% non-fat-dried milk in PBS for 1 h. Immunostaining for p-SMAD2 (1:1000) and Snail (1:1000) was performed overnight at 4 °C in 2% BSA in PBS followed by 1 h incubation with Goat anti-Rabbit IgG (H + L), Alexa Fluor 488 conjugate (1:2000) as secondary antibody. The cilium was stained with the antibody specific for acetylated-α-tubulin (1:2000) and Goat anti-Mouse IgG (H + L) Alexa Fluor 594 conjugate (1:3000) or Alexa Fluor 488 conjugate (1:3000) as secondary antibody. F-actin was visualized using Phalloidin-Atto 594 (1:1500). Immunofluorescence slides were mounted with Vectashield containing DAPI after secondary antibody incubation and pictures were taken on the Leica DM5500 B microscope.

### Statistical analysis

Results are expressed as mean ± SD. Differences between one treatment group and their controls were tested using two-tailed Student’s *t* tests. One- or two-way analysis of variance (ANOVA) was used, when three or more groups were compared, followed by post hoc Fisher’s LSD multiple comparison, if the overall ANOVA *F* test was significant. *P* < 0.05 was considered to be statistically significant.

## Results

### Fluid shear stress increases SMAD2/3 activity and alters expression of epithelial-to-mesenchymal transition (EMT) markers

**Fig. 1:**
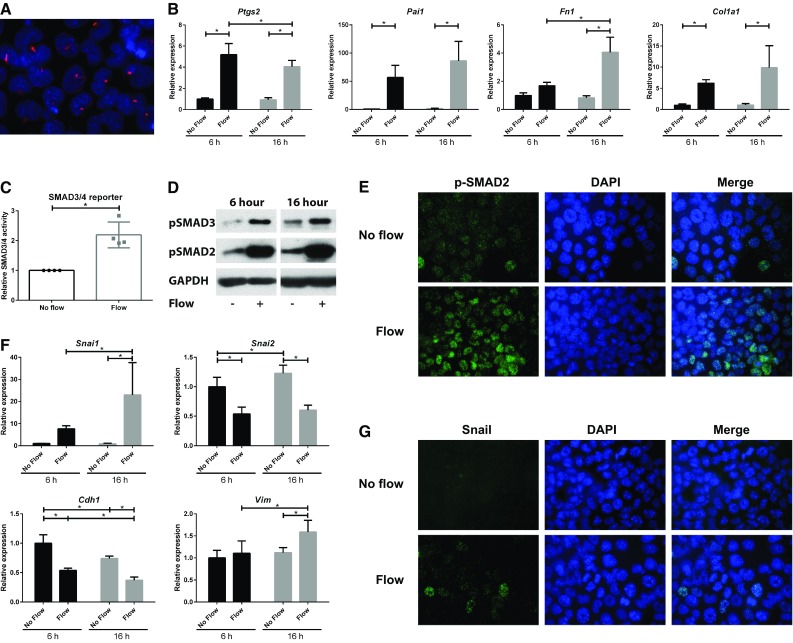
Activation of SMAD2/3 signaling by fluid flow in ciliated PTECs. **a** Serum starvation induces cilia formation in proximal tubular epithelial cells (PTECs). Cilia are visualized using anti-acetylated α-tubulin antibodies (*red*) and nuclei are stained with DAPI (*blue*). **b** Relative expression of *Ptgs2* (COX2) and *Pai1* (plasminogen activator inhibitor 1; *Serpine1*), *Fn1* (EDA region; fibronectin) and *Col1a1* (collagen, type I, alpha 1) is increased upon fluid flow, as measured by quantitative PCR. Cone–plate induced fluid flow at *t* = 6 or 16 h; *Hprt* served as housekeeping gene to correct for cDNA input; data normalized to unstimulated PTECs at 6 h; *n* = 5 per condition; *P < 0.05 using two-way ANOVA. **c** SMAD3-SMAD4 (GACA_12_-Luciferase) transcriptional reporter activity was elevated, as measured upon 20 h of fluid flow stimulation. Data normalized to unstimulated PTECs (fold change); *n* = 4 per condition; *P < 0.05 using a two-tailed Student’s *t* test. **d** Western blot analysis of p-SMAD2 and p-SMAD3 shows increased phosphorylation upon 6 and 16 h fluid flow stimulation. GAPDH served as loading control. **e** Nuclear accumulation of p-SMAD2 (*green; t* = 6 h, IF). Nuclei are visualized with DAPI (*blue*). **f** Relative expression of *Snai1* (Snail) and *Vim* (vimentin) is increased, while relative expression of *Snai2* (Slug) and *Cdh1* (E-cadherin) is reduced in PTECs stimulated with fluid flow, as measured by quantitative PCR. Cone–plate induced fluid flow at *t* = 6 or 16 h; *Hprt* served as housekeeping gene to correct for cDNA input; data normalized to unstimulated PTECs at 6 h; *n* = 5 per condition; *P < 0.05 using two-way ANOVA. ** g** Nuclear accumulation of Snail (*green; t* = 6 h, IF). Nuclei are visualized with DAPI (*blue*)

To study fluid flow-induced cellular alterations, ciliated proximal tubular epithelial cells (PTEC; Fig. 1a) were exposed to a fluid shear stress of ~1.9 dyn/cm^2^, using a cone–plate device. After 6 or 16 h fluid flow exposure, gene expression was analyzed using quantitative PCR (qPCR). We first confirmed increased mRNA levels of *Ptgs2*, the gene encoding cyclo-oxygenase 2 (COX2), a known flow responsive gene (Fig. 1b) [42].

A crucial step in TGF-β signaling is the activation and translocation of phosphorylated SMAD2 and 3 (p-SMAD2/3) to the nucleus to induce the expression of SMAD3 target genes, i.e., *Pai1, Fn1*, and *Col1a1*. Indeed, expression of these genes was significantly increased upon fluid flow at both time points indicating activation of this pathway (Fig. 1b). Increased TGF-β signaling was confirmed using the SMAD3-SMAD4 transcriptional reporter CAGA_12_-Luc (Fig. 1c) [37]. In addition, elevated levels of p-SMAD2 and p-SMAD3 were detected by western blot analysis (Fig. 1d). Furthermore, nuclear translocation of p-SMAD2 was observed by immunofluorescence microscopy (Fig. 1e). Similar responses were seen in *Pkd1* ^−/−^ PTECs, in which expression of the *Pkd1*-gene, encoding a potential flow sensor [12], is disrupted (Supplementary Material 1, Fig. S1). While expression of the SMAD2/3 targets was clearly elevated upon fluid flow, expression of the canonical and non-canonical Wnt targets (*Ccnd1, Axin2, Birc5* (*Survivin*), *Lin7a, Ppard, Glis2, Insc*), and hedgehog targets (*Gli1, Gli2, Gli3*) was virtually not altered (Supplementary Material 1, Fig. S2).

Increased SMAD2/3 activity often is associated with dedifferentiation and EMT-like processes, regulated via the transcription factors Snail and/or Slug [43]. Indeed, we also observed differential expression of EMT marker genes *Snai1, Snai2, Cdh1*, and *Vim*, encoding the proteins Snail, Slug, E-cadherin, and vimentin, respectively (Fig. 1f). mRNA levels of *Snai1* and *Vim* were increased while expression of the epithelial marker *Cdh1* was decreased. Even more, nuclear accumulation of Snail was detected upon fluid flow (Fig. 1g). Similar flow responses were seen in *Pkd1* ^−/−^ PTECs (Supplementary Material 1, Fig. S1). Interestingly, while Snail and Slug frequently show co-expression [44], our data clearly show fluid flow-induced downregulation of *Snai2*, the gene encoding the protein Slug. These data suggest that in this context Snail is responsible for the expression of mesenchymal markers and for repression of the epithelial E-cadherin gene.

### TGF-β/activin-induced dose- and time-dependent activation of SMAD2/3 signaling

**Fig. 2:**
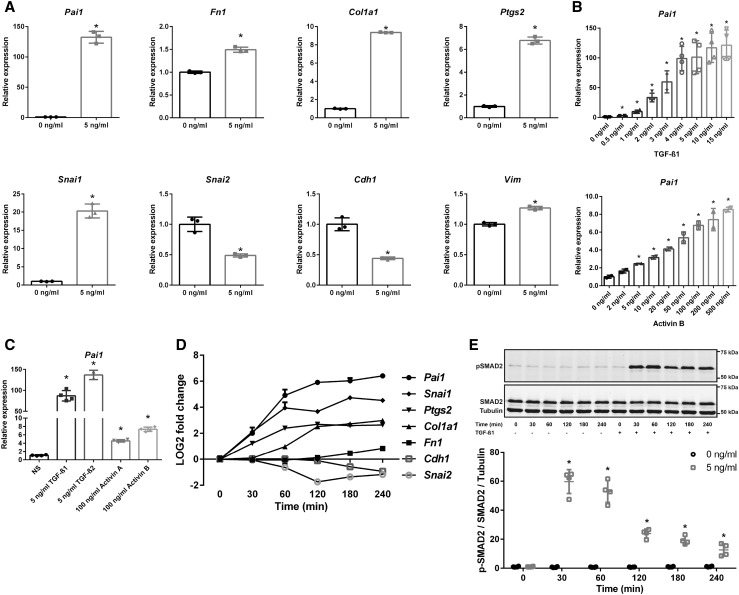
Dose- and time-dependent activation of SMAD2/3 signaling by TGF-β and activin. **a** Increased expression of *Pai1, Fn1, Col1a1, Ptgs2, Snai1*, and *Vim*, and reduced expression of *Snai2* and *Cdh1*, upon stimulation with 5 ng/ml TGF-β1 (*n* = 3, *t* = 4 h). **b** A dose response experiment shows increased sensitivity of *Pai1* mRNA expression for TGF-β1 (*n* = 4) compared to activin B (*n* = 2) stimulation (*t* = 4 h). **c** *Pai1* expression shows stronger induction upon TGF-β1 or TGF-β2, compared to activin A or activin B (*n* = 4 per condition, *t* = 4 h). **d** Time response (0–240 min) of target genes upon 5 ng/ml TGF-β1 stimulation (*n* = 2). Expression was significantly different (*P* < 0.05; one-way ANOVA) with 5 ng/ml TGF-β1 stimulation compared to non-treated controls for *Pai1, Ptgs2*, and *Snai1* at 30 min; for *Col1a1* and *Snai2* at 60 min; for *Fn1* and *Cdh1* at 180 min. **e** Representative western blot of p-SMAD2 and SMAD2 upon 5 ng/ml TGF-β1 stimulation (time response of 0–240 min). Tubulin served as loading control. For quantification, p-SMAD2 levels were corrected for total SMAD2 and tubulin levels (*n* = 4). Relative mRNA expression was measured by quantitative PCR, where *Hprt* served as housekeeping gene to correct for cDNA input (**a**–**d**). *P < 0.05 compared to non-treated control using a two-tailed Student’s *t* test. *NS* not stimulated control

Next, we wondered whether TGF-β or activin, the cytokines that can induce SMAD2/3 phosphorylation, could generate the same gene expression pattern as fluid flow. Indeed, the same expression profile was observed upon TGF-β1 stimulation, including upregulation of *Snai1* and down-regulation of S*nai2* (Fig. 2a). A dose response curve and comparison of the cytokines indicated that the cells were more sensitive to TGF-β1 or -β2 as to activin A or B (Fig. 2b, c).

A time course experiment showed that expression of the canonical SMAD2/3 target, *Pai1*, was already significantly induced after 30 min of TGF-β1 stimulation (Fig. 2d), while *Col1a1* followed at 60 min and *Fn1* at 180 min. Surprisingly, *Ptgs2* and *Snai1* expression were also induced after 30 min (Fig. 2d) suggesting that these genes could be SMAD2/3 targets as well, because SMAD2 is phosphorylated within 30 min after TGF-β stimulation (Fig. 2e). The downregulated genes, *Snai2* and *Cdh1*, showed a significant decrease in expression starting at 60 and 180 min, respectively. Our data indicate that, *Pai1, Ptgs2, Snai1*, and *Col1a1* are early responsive genes upon TGF-β stimulation, while *Fn1* is a late responsive gene.

### Altered expression of TGF-β/activin ligands and receptors upon fluid flow

**Fig. 3:**
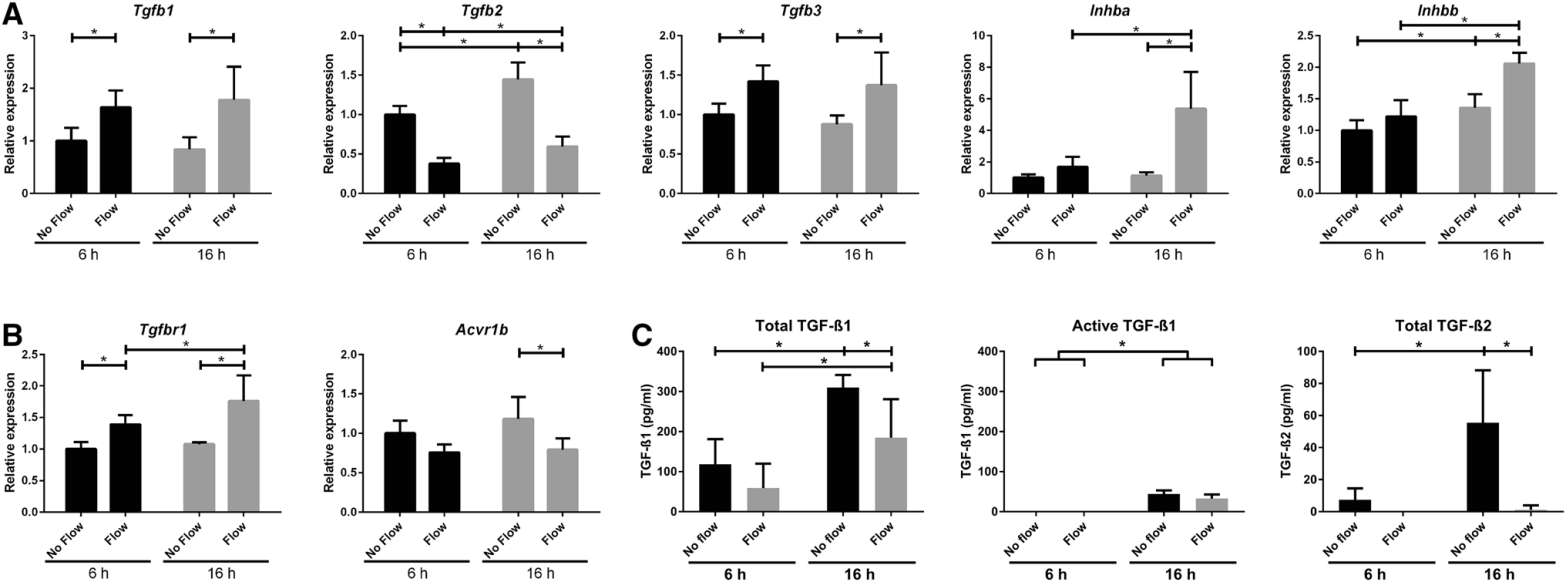
Fluid flow altered expression of the TGF-β and activin ligands as well as their receptors *Alk5* and *Alk4*. **a** Relative expression of *Tgfb1, Tgfb2, Tgfb3, Inhba, Inhbb* and **b** *Tgfbr1* (*Alk5*) and *Acvr1b* (*Alk4*) mRNA in PTECs upon fluid flow. Cone–plate-induced fluid flow at *t* = 6 or 16 h; qPCR, *Hprt* served as housekeeping gene to correct for cDNA input; data normalized to unstimulated PTECs at 6 h; *n* = 5 per condition; *P < 0.05 using two-way ANOVA, followed by post hoc Fisher’s LSD multiple comparison. **c** Levels of TGF-β1 (total and active) and TGF-β2 (total) in the medium of PTECs collected after 6 or 16 h fluid flow. TGF-β3 and active TGF-β2 levels in medium and TGF-β1, 2, and 3 levels in cell lysates were below the detection limit. Cone–plate-induced fluid shear stress; TGF-β levels measured by ELISA; *n* = 5 per condition; *P < 0.05 using two-way ANOVA, followed by post hoc Fisher’s LSD multiple comparison

Activation of SMAD2/3 is largely regulated via TGF-β or activin receptor complexes, upon binding of their respective ligands [23]. Therefore, expression of the genes coding for ligands TGF-β1, -2, and -3 or coding for activin A and B (i.e., *Inhba* and *Inhbb*) was measured by qPCR. Our data show a significant flow-induced increase in expression of *Tgfb1 and Tgfb3* as well as *Inhba* and *Inhbb* upon 16 h fluid flow stimulation, while this trend was already visible upon 6 h fluid flow (Fig. 3a). At both time-points *Tgfb2* transcript levels were significantly decreased.

Next, we measured protein levels of TGF-β ligands in the medium collected after 16 h fluid shear (Fig. 3c). Total TGF-β1 and TGF-β2 levels were significantly decreased upon fluid flow in the medium, while active TGF-β1 was lower, but not significantly changed by flow. TGF-β levels at 16 h fluid shear stress were significantly higher compared to 6 h (Fig. 3c), suggesting there is production of latent TGF-β protein in time. Active and total TGF-β3 as well as active TGF-β2 were below detection levels (data not shown). In cell lysates, total TGF-β levels were mainly below detection level, except TGF-β1 levels of a few of the measured fluid flow samples at 16 h (data not shown).

We also analyzed expression of the different receptors. The type-I receptor ALK4 or ALK5 is recruited and trans-phosphorylated by their specific type-II receptor upon binding of activin or TGF-β ligands, respectively. Expression of *Alk5* (*Tgfbr1*) transcript was significantly increased upon fluid flow, while *Alk4* (*Acvr1b*) was decreased (Fig. 3b). Expression of the type-II receptors did not change and *Alk7* (*Acvr1c*) was not expressed in PTECs (data not shown). Similar shear stress responses were seen in *Pkd1* ^−/−^ PTEC cells (Supplementary Material 1, Fig. S3). Overall, our data are inconclusive about the role of the ligands and receptors during fluid flow-induced SMAD2/3 activation. Nevertheless, increased SMAD2/3 activation could be the result of receptor activation.

### Shear stress-induced SMAD2/3 target gene expression is flow-rate dependent, but partially cilia independent

**Fig. 4:**
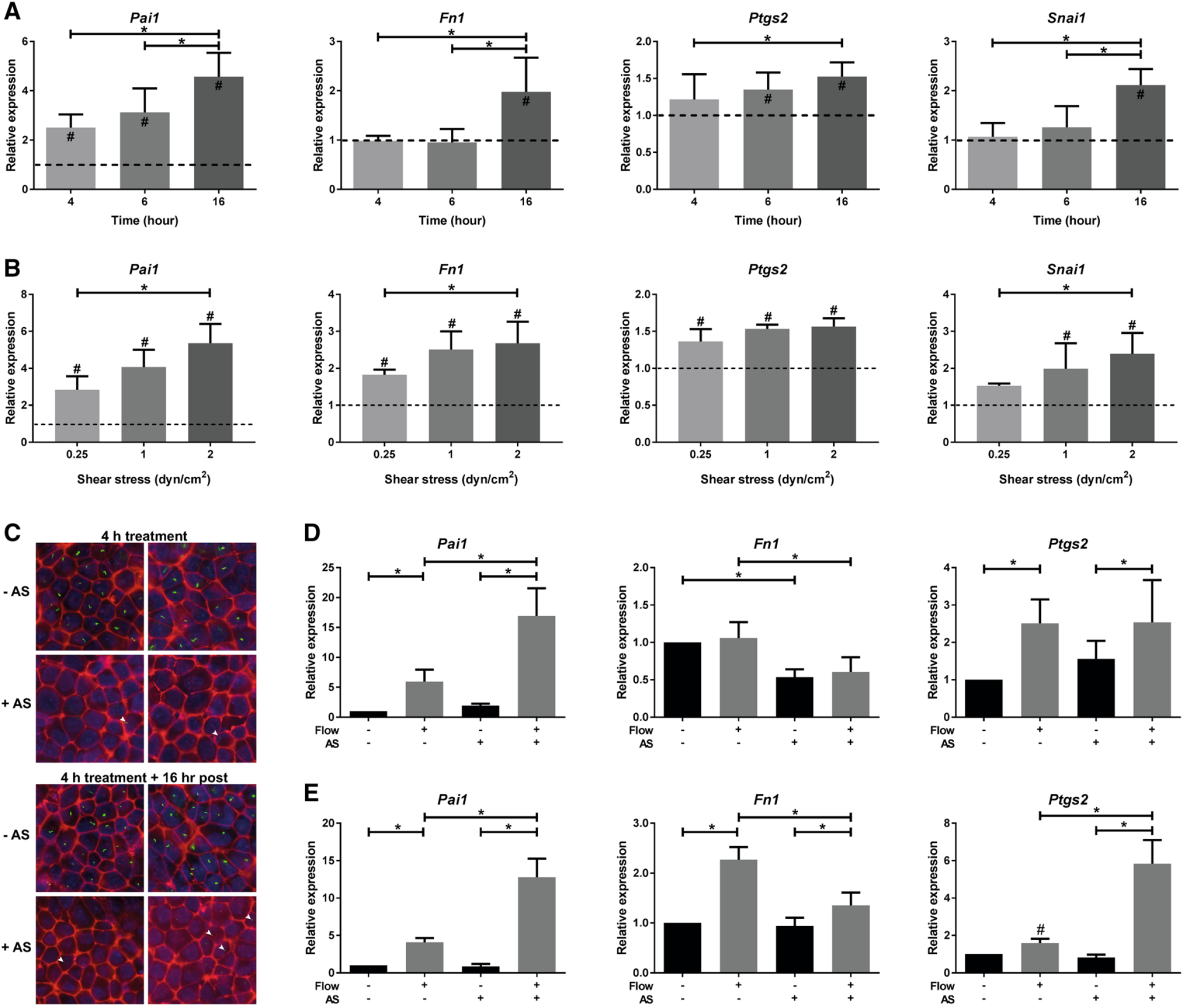
Shear stress-induced SMAD2/3 target gene expression in PTECs is flow rate dependent, but partially cilia independent. **a, b** Relative expression (fold change) of *Pai1, Fn1, Ptgs2*, and *Snai1* is gradually increased in time (**a** *t* = 4, 6 or 16 h; *n* = 5 per condition) and upon increasing flow rates in PTECs (**b** 0.25, 1.0 or 2.0 dyn/cm^2^; *n* = 3 per condition), as measured by quantitative PCR. Parallel plate flow chamber induced fluid shear stress; *Hprt* served as housekeeping gene to correct for cDNA input; data were normalized to static controls (fold change). ^#^Significant difference compared to unstimulated control (*dashed line*) or *significant difference between treatment groups (*P* < 0.05 by two-way ANOVA, followed by post hoc Fisher’s LSD multiple comparison). **c** To remove cilia, cells were treated with 50 mM ammonium sulfate (AS) for 4 h, followed by 16 h post incubation. Cilia were visualized by IF using anti-acetylated α-tubulin antibodies (*green*), F-actin using phalloidin antibodies (*red*) and nuclei were stained with DAPI (*blue*). Control cells clearly showed cilia staining, while AS-treated cells only showed weak or stunted cilia staining (*arrowhead*). **d, e** Relative expression of *Pai1, Fn1*, and *Ptgs2* is increased upon 6 (**d**) or 16 (**e**) h fluid shear stress in controls and cells treated with 50 mM ammonium sulfate (AS), as measured by quantitative PCR. Parallel plate flow chamber induced fluid shear stress at 2.0 dyn/cm^2^ in PTECs; *n* = 5 per condition; *Hprt* served as housekeeping gene to correct for cDNA input; data were normalized to static controls (fold change). *P < 0.05 by two-way ANOVA, followed by post hoc Fisher’s LSD multiple comparison. ^#^Significantly altered expression by flow versus no flow (*P* < 0.05) using a two-tailed Student’s *t* test

We subsequently performed experiments using a parallel plate flow chamber [34, 35] and confirmed the fluid shear-induced expression of SMAD2/3 target genes and EMT markers. With this device, fluid shear stress-induced SMAD2/3 target gene expression is lower compared to the cone–plate flow system and for several genes only the 16 h responses are significant (Fig. 4a). Likely, this can be attributed to the larger volume of culture medium that is circulated in the parallel plate flow system, thereby diluting the concentration of ligands produced by the cells. A flow rate response curve showed a gradual increase of SMAD2/3 target gene expression (Fig. 4b). Surprisingly, removal of cilia by ammonium sulfate (AS) further enhanced the fluid flow-induced expression of *Pai1* and *Ptgs2* (Fig. 4c–e), though *Fn1* induction was lower. Our data suggest that cilia do not fully control the SMAD2/3 response in PTECs, indicating a complex fluid shear stress response, where yet unidentified mechano-sensors might be involved.

### Shear stress-induced SMAD2/3 activation can be largely blocked by ALK4/5/7 inhibitors

**Fig. 5:**
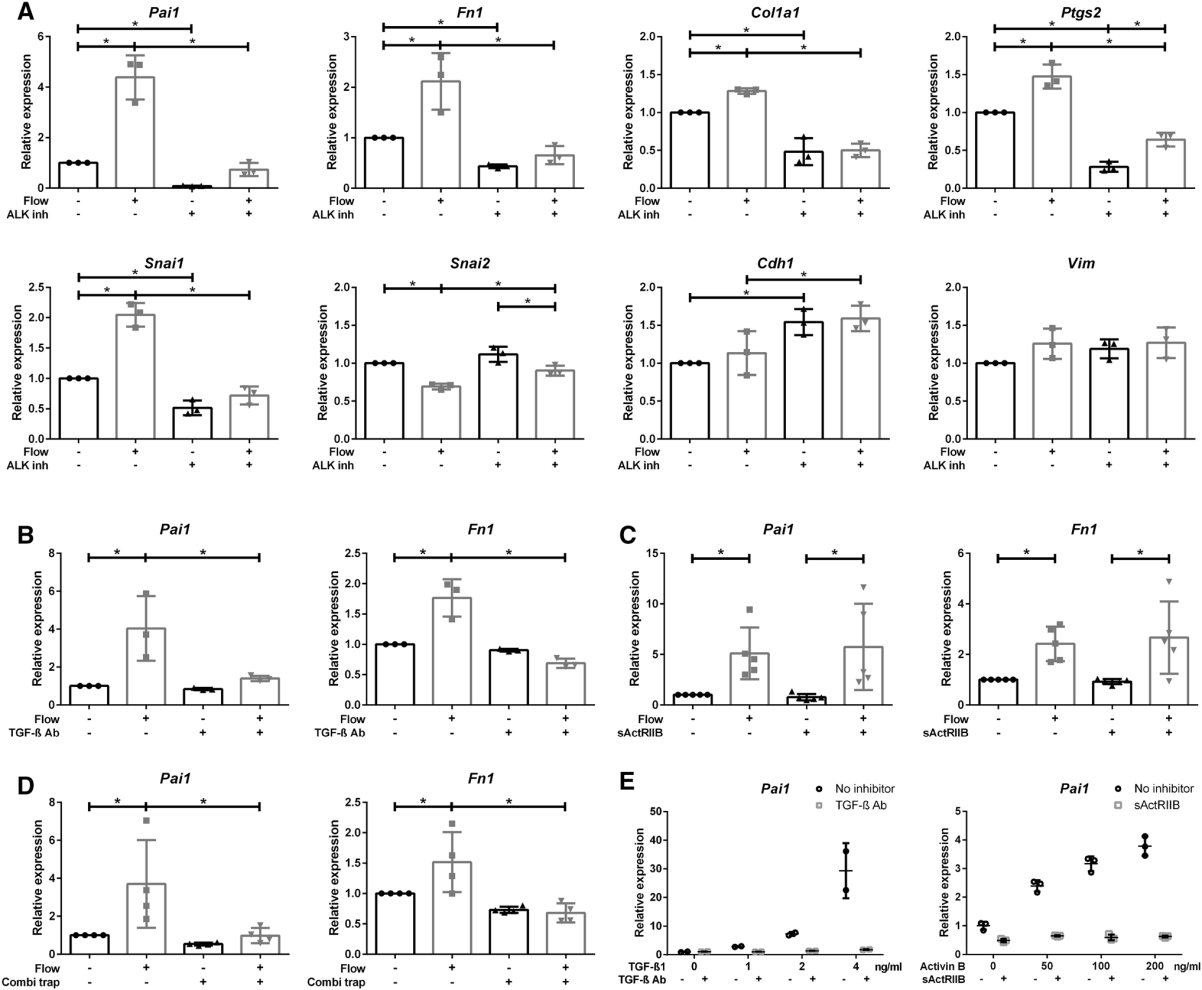
ALK4/5/7 inhibitor and TGF-β neutralizing antibodies, but not sActRIIB-Fc, effectively block SMAD2/3 signaling upon fluid flow stimulation. **a** ALK4/5/7 inhibitor (LY-364947; *n* = 3) significantly reduces baseline and fluid flow increased expression of *Pai1, Fn1, Col1a1, Ptgs2, and Snai1* while the expression of *Snai2* is less decreased. **b** TGF-β neutralizing antibodies (TGF-β Ab; *n* = 3) inhibited fluid flow-induced expression of SMAD2/3 target genes (*Pai1 and Fn1*), while **c** soluble activin type-IIB-receptor fusion protein (sActRIIB-Fc; *n* = 5) did not. **d** Combining TGF-β Ab with sActRIIB-Fc (*n* = 4) did not further increase the inhibitory effect. **e** TGF-β1 (*n* = 2) or activin B (*n* = 3) ligand-induced *Pai1* expression was effectively inhibited by TGF-β Ab or sActRIIB-Fc, respectively (*t* = 4 h). Parallel plate flow chamber induced fluid shear stress in PTECs at *t* = 16 h (**a**–**d**); qPCR, *Hprt* served as housekeeping gene to correct for cDNA input; data normalized to unstimulated controls (fold change); *n* = 2–5 per condition as indicated. *P < 0.05 by two-way ANOVA, followed by post hoc Fisher’s LSD multiple comparison.* ALK inh* ALK4/5/7 inhibitor (LY-364947),* Combi trap* combined ligand traps (TGF-β Ab and sActRIIB-Fc)

To interfere with receptor activation, an ALK5 inhibitor (LY-364947) that abrogates ALK4, ALK5, and ALK7 kinase activity [45–48] was added to the medium. Cells were pre-incubated with the inhibitor and stimulated by fluid flow for 16 h using the parallel plate flow chamber. Expression of the SMAD2/3 target genes (*Pai1, Fn1, and Col1a1*), but also *Ptgs2* was strongly reduced by the inhibitor in samples with and without flow, as shown for LY-364947 (Fig. 5a). However, expression was not entirely blocked and a very mild flow response can still be appreciated, which is only significant for *Ptgs2*. These effects were confirmed using another ALK4/5/7 inhibitor, SB431542, which resulted in a similar pattern (data not shown). TGF-β1-induced expression of SMAD2/3 targets (*Pai1, Fn1*, and *Ptgs2*) was similarly blocked by the ALK4/5/7 inhibitor (Supplementary Material 1, Fig. S4a).

Interestingly, the increased expression of *Snai1* (encoding Snail) was also strongly reduced with the ALK4/5/7 inhibitor, while the expression of *Snai2* (encoding Slug) was less reduced (Fig. 5a). Expression of the Snail target *Cdh1* is increased with the ALK4/5/7 inhibitor, but not altered by fluid flow, probably caused by the low induction of *Snai1*. These data suggest that, besides *Pai1, Fn1, Col1a1*, and *Ptgs2*, also expression of *Snai1* is largely mediated via SMAD2/3 signaling.

### Shear stress-induced SMAD2/3 activation is abrogated by TGF-β neutralizing antibodies, but not by an activin ligand trap

To discriminate between ALK5 and ALK4 activation and to prevent ligand binding, TGF-β neutralizing antibodies (TGF-β Ab) or soluble activin receptor-IIB fusion proteins (sActRIIB-Fc) that functions as ligand trap for activin have been added to the medium of fluid flow-stimulated cells and controls [49–51]. Fluid shear stress-induced expression of SMAD3 target genes, *Pai1* and *Fn1*, was significantly decreased with TGF-β Ab, but not with sActRIIB-Fc (Fig. 5b, c). In control samples, i.e., static cells stimulated with exogenous TGF-β or activin, the responses were blocked, proving the efficacy of the inhibitors (Fig. 5e, Supplementary Material 1, Fig. S4c, d). Also, the combination of TGF-β Ab and sActRIIB-Fc showed a similar decrease in fluid shear-induced expression of SMAD3 target genes as TGF-β neutralizing Ab alone (Fig. 5d). From this, we conclude that shear stress-induced SMAD2/3 activity was strongly reduced by the ALK4/5/7 receptor kinase inhibitor and by TGF-β neutralizing Ab, but was not affected by the ligand trap sActRIIB-Fc, indicating a major role for TGF-β in the fluid shear stress response.

### SMAD2/3 regulated gene expression is modulated by MEK1/2

**Fig. 6:**
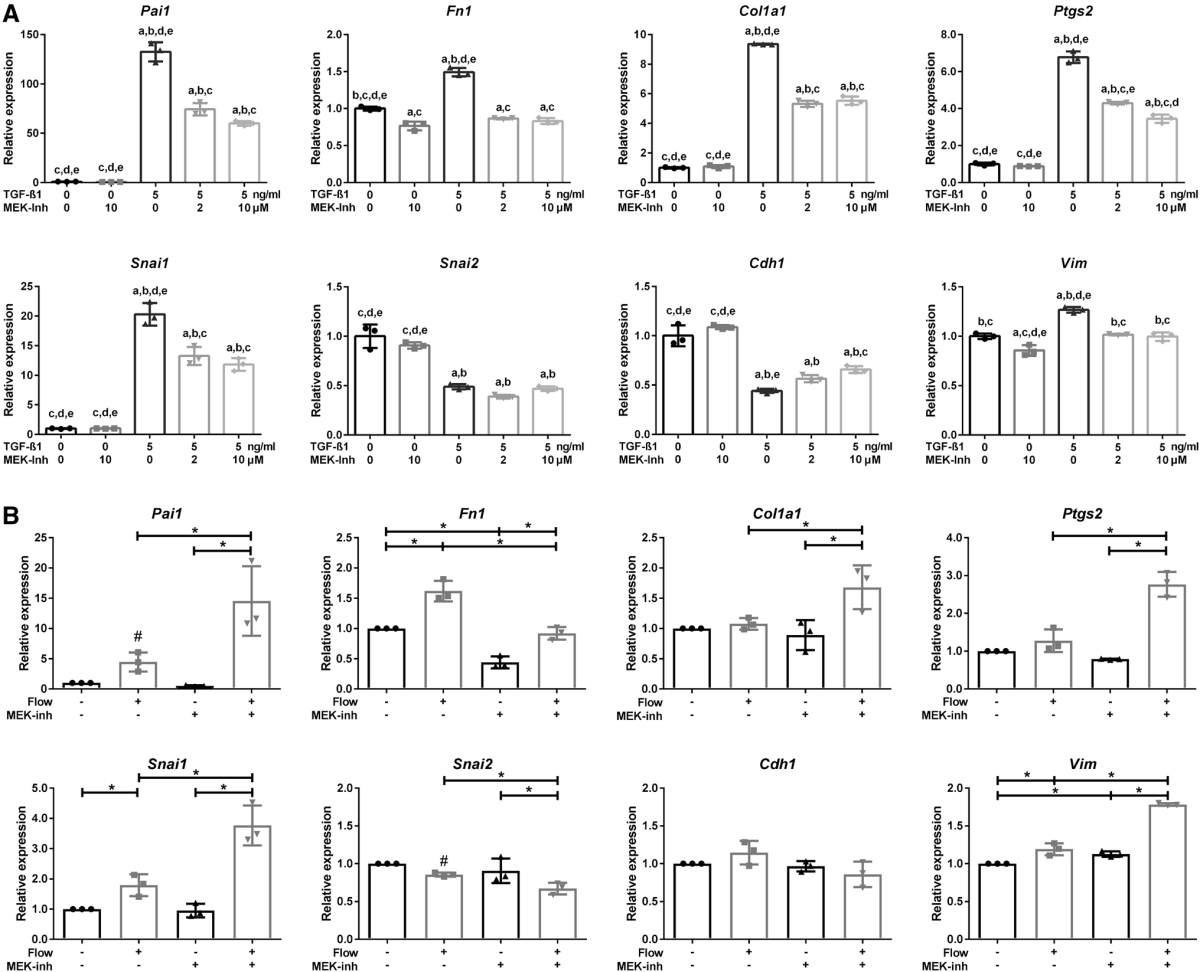
MEK inhibition modulates fluid shear-induced and TGF-β-stimulated expression of SMAD2/3 target genes. **a** MEK inhibition (Trametinib, GSK1120212) reduces TGF-β1 increased expression of *Pai1, Fn1, Col1a1, Ptgs2, Snai1, and Vim*, while expression of *Cdh1* is less decreased. *Snai2* expression was not significantly changed upon MEK inhibition. Relative mRNA expression measured at *t* = 4 h; *n* = 3; *Hprt* served as housekeeping gene to correct for cDNA input; data normalized to unstimulated controls. Significant difference (*P* < 0.05) by one-way ANOVA: ^a^compared to 0 ng/ml TGF-β1, ^b^compared to 0 ng/ml TGF-β1 + 10 µM MEK-inh, ^c^compared to 5 ng/ml TGF-β1, ^d^compared to 5 ng/ml TGF-β1 + 2 µM MEK-inh or ^e^compared to 5 ng/ml TGF-β1 + 10 µM MEK-inh. **b** MEK inhibition (10 µM Trametinib) reduces fluid flow-increased expression of *Fn1*, while fluid flow increased expression of *Pai1, Col1a1, Ptgs2*, *Snai1*, and *Vim* is further elevated. Parallel plate flow chamber induced fluid shear stress in PTECs at *t* = 16 h; qPCR, *Hprt* served as housekeeping gene to correct for cDNA input; data normalized to unstimulated controls (fold change); *n* = 3 per condition. *P < 0.05 by two-way ANOVA, followed by post hoc Fisher’s LSD multiple comparison. ^#^Significantly altered expression by flow versus no flow (*P* < 0.05) using a two-tailed Student’s *t* test.* MEK-inh* MEK1/2 inhibitor (2 or 10 µM Trametinib, GSK1120212)

In addition to activation of SMAD2/3 signaling, TGF-β receptors can also activate MAPK/ERK signaling, which is able to modulate SMAD2/3 transcriptional activity [52, 53]. To interfere with MAPK/ERK signaling, an inhibitor was used that abolished MEK1/2 kinase activity, thereby preventing ERK1/2 phosphorylation [54]. Indeed, a MEK1/2 inhibitor (2 or 10 µM Trametinib, GSK1120212) reduced TGF-β1-induced expression of the canonical SMAD3 targets genes (*Pai1, Fn1*, and *Col1a1*) as well as *Ptgs2* (Fig. 6a, Supplementary Material 1, Fig. S4b). Furthermore, expression of *Snai1* and *Vim* was reduced and, correspondingly, expression of the epithelial marker *Cdh1* was less decreased (Fig. 6a). Surprisingly, fluid flow induction of *Pai1, Col1a1, Ptgs2, Snai1*, and *Vim* was further enhanced with the MEK1/2 inhibitor, while the baseline and fluid flow-induced *Fn1* expression was lower during MEK inhibition (Fig. 6b, Supplementary Material 1, Fig. S5a). This cannot be explained by a low TGF-β dose that is produced by PTECs during fluid flow, because SMAD2/3 target gene expression was also reduced by the MEK1/2 inhibitor during low dose (0.25–2 ng/ml) TGF-β1 stimulation (Supplementary Material 1, Fig. S5b). This suggests a complex regulation of SMAD2/3 target genes during shear stress, which is differently modulated by MEK1/2 than in static cells upon exogenous TGF-β stimulation.

## Discussion

Fluid flow stimulation has been used to study mechanical shear stress in endothelial cells and osteoblasts for many years [55, 56]. In the last decade, this was extended to epithelial cells [57]. Even more, several inherited renal diseases are a consequence of dysfunctional primary cilia, with Polycystic Kidney Disease (PKD) as most prominent example [15]. In PKD, a gradual decline of functional nephrons results in compensatory hyperfiltration and increased shear stress in the remaining nephrons. In contrast, in a nephron that becomes cystic, tubular dilation will result in reduced fluid flow.

Our results show fluid flow induced TGF-β/ALK5-mediated signaling, SMAD2/3 phosphorylation, and target gene expression in PTECs. This can be inhibited by an ALK4/5/7 inhibitor, which indicates that, like in endothelial cells [21], autocrine signaling is involved. Even more, TGF-β neutralizing antibodies block the flow response, similar to the inhibition of exogenously added active TGF-β1 under static conditions. In contrast, sActRIIB-Fc, a ligand trap that sequesters activin did not block the flow response, while the effect of exogenously added activin under static conditions was completely blocked. A previous study reported that fluid shear stress dynamically regulated TGF-β gene expression and SMAD3 activation, depending on the magnitude of fluid shear [22, 26]. In the current study, we confirmed in ciliated cells that the flow-induced response was depending on the magnitude of shear (0.25–2 dyn/cm^2^). In addition, cells were grown on a collagen type-I extracellular matrix, which is known to influence the mechanical forces that are sensed by cells [58]. Upon deciliation of renal epithelial cells by ammonium sulfate [36], however, the fluid flow response remained (Fig. 4c–e). Moreover, expression of early responsive genes was enhanced upon deciliation, suggesting that to a certain extent primary cilia restrain TGF-β/ALK5 signaling. However, expression of the late-responsive gene *Fn1* was less increased, indicating a complex regulation of the shear-induced SMAD2/3 response in PTECs. These data also suggest involvement of yet unidentified mechano-sensors located at other parts of the plasma membrane.

The flow response could be inhibited by the ALK4/5/7 inhibitor and TGF-β neutralizing antibodies, and TGF-β1 and -3 mRNA levels were increased by fluid flow. However, latent TGF-β protein levels were lower in culture medium of shear stress-treated PTECs. It is conceivable that under flow conditions TGF-β processing and binding of the active ligand is enhanced, and therefore local effects are stronger. For example, growth factor shedding and autocrine signaling via the intercellular space upon mechanical stimulation of epithelial cells, had previously been shown for HB-EGF [59]. However, this explanation is less likely, since latent TGF-β is excreted fast after production [60] and we showed accumulation in the medium (Fig. 3c, Supplementary Material 1, Fig. S3c). An alternative explanation could be associated to enhanced sensitivity for TGF-β, since we observed increased mRNA expression of the ALK5 receptor upon fluid flow. In addition, increased flow-induced apical endocytosis might play a role, as recently published for PTECs [61].

Also *Pkd1* ^−/−^ PTECs showed increased SMAD2/3 activation upon fluid flow treatment, suggesting that polycystin-1, the protein encoded by *Pkd1*, is not directly involved in this response. Nevertheless, the response is slightly but significantly stronger in *Pkd1* ^−/−^ PTECs (Supplementary Material 1, Fig. S1k). Higher responsiveness in *Pkd1* deficient vascular smooth muscle cells and murine embryonic fibroblast cells by TGF-β acting stimuli have been reported previously [62]. Alternatively, higher levels of TGF-β2, measured in the medium of *Pkd1* ^−/−^ PTECs during fluid flow (Supplementary Material 1, Fig. S3), might be responsible for the enhanced response, which seems more likely given the fact that exogenous TGF-β1 or activin B stimulation of static cells did not induce an enhanced response in *Pkd1* ^−/−^ PTECs (data not shown). In ADPKD kidneys, somatic inactivation of *PKD1* or *PKD2* is a critical step in cyst formation [63, 64]. ADPKD is a progressive disease in which the number and size of cysts increase in time accompanied by increased fibrosis. Consequently, the landscape of cells with and without polycystin expression, and nephrons with increased, reduced or no-flow conditions alters in time as well. This is related to hyperfiltration, tubular dilation or tubular obstruction. Increased fluid shear stress and TGF-β signaling will occur in the nephrons compensating the functional loss. So, the elevated response of *Pkd1* ^−/−^ cells upon fluid flow is most relevant in the early phase upon disruption of the gene and during hyperfiltration in the remaining nephrons. Of course, when nephrons are dilated and the fluid flow itself is altered, the flow response will change again and probably diminish.

In ADPKD nuclear accumulation of SMAD2/3 in cystic epithelial cells and in interstitial fibroblasts, as well as elevated expression of target genes, points to a role of SMAD2/3-regulated signaling in epithelial dedifferentiation and fibrosis [24, 27, 65]. In cystic epithelial cells of end-stage PKD, TGF-β1, and SMAD2/3 signaling was upregulated and associated with renal EMT and renal fibrosis [66]. Paradoxically, genetic disruption of the ALK5 receptor in renal epithelial cells did not affect cyst formation/growth and only slightly reduced expression of SMAD2/3 target genes, while the activin ligand trap, sActRIIB-Fc, significantly slowed down PKD progression in mice [65]. This indicates that for cyst formation and PKD progression, the tissue context and the different cell types involved are critical.

Increased SMAD2/3 activity is often associated with dedifferentiation and EMT-like processes, which is needed for epithelial cell plasticity and homeostasis during tissue development, maintenance, and repair [67]. TGF-β can induce Snail expression, which is known to directly repress epithelial markers like E-cadherin [68], and TGF-β can induce expression of markers of the mesenchymal phenotype, including vimentin [69]. Indeed, we observed fluid flow induced expression and nuclear accumulation of Snail, as well as increased vimentin and reduced E-cadherin expression. Interestingly, while Snail and the highly homologous protein Slug are frequently co-expressed [44], *Snai2*, the gene encoding Slug, is clearly down-regulated upon fluid flow. Therefore, we conclude that in this context Snail, but not Slug, is responsible for the expression of mesenchymal markers and for repression of the epithelial E-cadherin gene.

In renal epithelial cells, TGF-β-induced expression of canonical SMAD2/3 targets and EMT markers is the consequence of both SMAD2/3 and ERK1/2 activation, since upstream ALK4/5/7 and MEK1/2 inhibitors both reduce but not completely block the ligand-induced response (Fig. 6, Supplementary Material 1, Fig. S4). While the fluid flow-induced response was largely blocked by the ALK4/5/7 inhibitor, the MEK inhibitor, however, further enhanced expression of *Pai1, Col1a1, Ptgs2, Snai1*, and *Vim*, though *Fn1* expression was less increased (Figs. 5, 6). It is well known that a cross-talk between the canonical (SMAD2/3) and the non-canonical (MAPK/ERK) TGF-β pathways take place at different levels and via direct or indirect pathways [70]. These data support a previous study describing antagonistic interactions between TGF-β1-mediated expression of fibrogenesis genes and the cAMP/PKA pathway. On the one hand, cAMP/PKA promoted TGF-β1 production and expression of target genes (including collagen-I and fibronectin) in MDCK cells and an embryonic kidney cyst model, being inhibited by an ALK5-inhibitor. On the other hand, upon addition of TGF-β1 to the culture medium of MDCK cells, fibronectin expression is negatively regulated upon cAMP treatment, via ERK1/2 [71]. A direct link between TGF-β and MAPK/ERK signaling, is the phosphorylation of ShcA by the activated TGF-β receptor complex [52]. ShcA competes with SMAD2/3 for binding to the TGF-β receptor, and stabilizes the TGF-β receptor complexes in caveolae, where it activates MAPK/ERK signaling [72]. Consequently, reduced ShcA expression results in increased levels of TGF-β receptor complexes in clathrin-coated pits, leading to enhanced SMAD2/3 activation. Alternatively, activated ERK1/2, by TGF-β/ALK5 or by other growth factors, can mediate phosphorylation of the regulatory SMADs (R-SMAD) as well as phosphorylation of the SMAD2/3 linker region, which modulate transcriptional activity of the SMAD complex [53, 73]. Because the multiple interactions between the MAPK/ERK and the TGF-β signaling cascades, the integration of these pathways is biological context dependent and complex, and therefore difficult to predict .

**Fig. 7:**
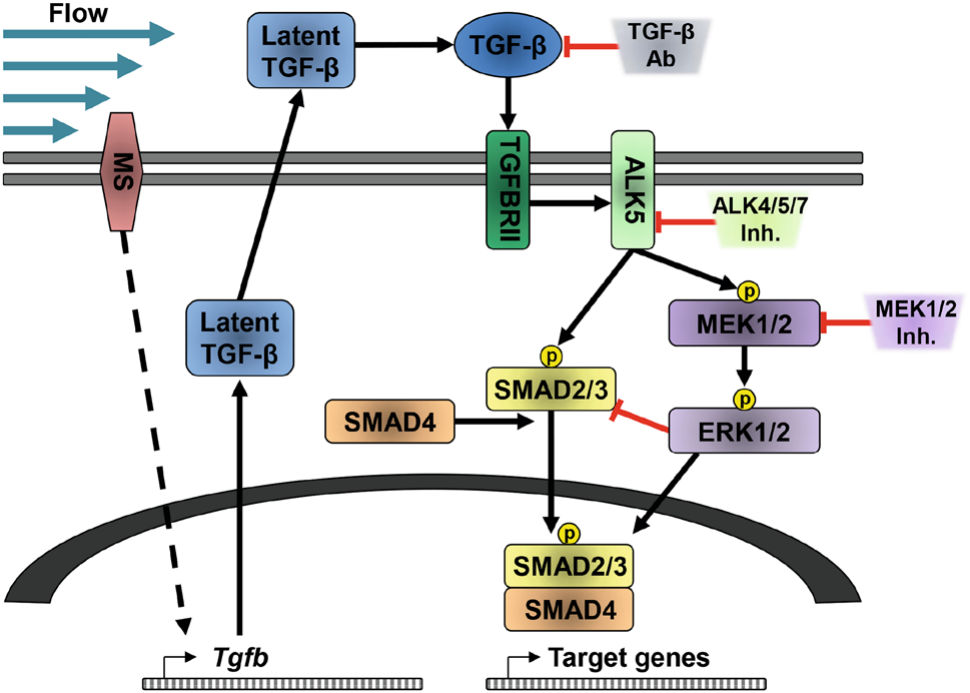
Schematic representation of the fluid shear stress response in PTECs. Fluid shear stress activates a yet unidentified mechano-sensor (MS) at the cell membrane. This leads to increased *Tgfb* mRNA expression. Upon activation, TGF-β will bind to TGFBRII, which recruits and activates ALK5. This is followed by SMAD2/3 phosphorylation, which recruits SMAD4 for nuclear entry, to enable target gene expression. Both the TGF-β-blocking antibodies (TGF-β Ab) and ALK4/5/7 inhibitors can block the shear stress response, inhibiting SMAD2/3 target gene expression. Alternatively, ALK5 can activate MEK1/2, a kinase known to phosphorylate ERK1/2 [72], which can be prevented by MEK inhibition. ERK1/2 can either enhance or repress SMAD2/3-mediated target gene expression, depending on the biological context and the cellular location where SMAD linker regions are phosphorylated by ERK1/2 [53, 73]. Cytoplasmic phosphorylation of SMAD2/3 by ERK1/2 can inhibit nuclear translocation, thereby restraining SMAD2/3 target gene expression. Upon MEK inhibition, more SMAD2/3 can translocate to the nucleus, thereby enhancing expression of early expressed SMAD2/3 target genes (*Pai1, Col1a1, Ptgs2*, and *Snai1*; see Fig. 2d). However, nuclear phosphorylation of SMAD2/3 linker regions increases the duration of their target gene expression, which is likely needed for fluid shear stress-induced expression of late genes (*Fn1*), since its induction is inhibited by the MEK inhibitor

Since *Fn1* expression was significantly increased by TGF-β1 at a later time point than the other target genes (Fig. 2d); it is likely that besides SMAD2/3, other transcription factors are involved in its mRNA expression. The literature indeed shows that *Fn1* expression was dependent on Snail or Slug expression and other transcription factors [74, 75]. Furthermore, to induce *Fn1* expression, it likely needs stabilized SMAD2/3 phosphorylation, which is achieved by ERK-mediated nuclear SMAD linker phosphorylation. This could be the explanation that TGF-β and fluid flow-induced *Fn1* expression is largely blocked upon MEK inhibition as proposed in the model depicted in Fig. 7.

The *Ptgs2* gene, encoding the COX2 protein, is widely used as a control for fluid flow. COX2-derived prostaglandins have been shown to stimulate cell proliferation, fluid secretion, and cyst formation in vitro, probably via increasing the cAMP levels. Furthermore, COX2-derived products show increased expression in animal models for PKD, and COX2 inhibitors showed minimal beneficial effect on PKD progression in rodent models [76, 77]. Our data show that *Ptgs2* expression is also TGF-β dependent and is regulated via shear stress in PTECs.

In conclusion, we found fluid shear stress-induced SMAD2/3 activation and target gene expression in ciliated and non-ciliated PTECs, suggesting that cilia are not critically inducing the shear stress-induced SMAD2/3 response of renal epithelial cells. Overall, our data indicate a complex response, in which yet unidentified mechano-sensors are involved. The response is dependent on autocrine TGF-β/ALK5-mediated signaling, but not on activin/ALK4. Under fluid shear stress conditions, the expression of most SMAD2/3 target genes is partially repressed by MAPK/ERK possibly via TGF-β/ALK5-mediated activation of MEK1/2 (Fig. 7) or via activation of this pathway by other yet unidentified autocrine (growth) factors. In renal (cystic) disease, compensatory hyperfiltration and increased shear stress might contribute to induced SMAD2/3 activation, a well-known factor in the control of epithelial cell plasticity and fibrosis.

## Electronic supplementary material

Below is the link to the electronic supplementary material.

Supplementary material 1 (PDF 1421 KB)

## Abbreviations

ADPKD: Autosomal dominant polycystic kidney disease
ALK: Activin-like kinase
AS: Ammonium sulfate
Cdh1: Cadherin-1
Col1a1: Collagen, type I, alpha 1
COX2: Cyclo-oxygenase-2
DPBS: Dulbecco’s phosphate-buffered saline
ELISA: Enzyme-linked immunosorbent assay
EMT: Epithelial-to-mesenchymal transition
ERK: Extracellular signal-regulated kinase
Fn1: Fibronectin 1
Hprt: Hypoxanthine–guanine phosphoribosyltransferase
MAPK: Mitogen-activated protein kinase
MEK: MAPK/ERK kinase
qPCR: Quantitative polymerase chain reaction
PAGE: Polyacrylamide gel electrophoresis
Pai1: Plasminogen activator inhibitor 1
PC: Polycystin
Pkd1: Polycystic kidney disease 1
PTEC: Proximal tubular epithelial cell
Ptgs2: Prostaglandin G/H synthase 2
RIPA: Radioimmunoprecipitation assay
sActRIIB-Fc: Soluble activin receptor-IIB fusion protein
SDS: Sodium dodecyl sulfate
SMAD: Mothers against decapentaplegic homolog
TBS: Tris-buffered saline
TGF-β: Transforming growth factorβ
TGF-β Ab: TGF-β neutralizing antibody
Vim: Vimentin
