# Supplementary material for: Fluid shear stress-induced TGF-β/ALK5 signaling in renal epithelial cells is modulated by MEK1/2
Source: Cell Mol Life Sci. 2017 Feb 6;74(12):2283–98. doi: 10.1007/s00018-017-2460-x (PMC5425503; doi:10.1007/s00018-017-2460-x)
Supplement: Supplementary file 1 — Supplementary material 1 (PDF 1421 KB) [file 18_2017_2460_MOESM1_ESM.pdf]

Fluid shear-stress induced TGF-β/ALK5 signaling in renal epithelial cells is modulated by MEK1/2

Cellular and Molecular Life Sciences

Steven J. Kunnen[1], Wouter N. Leonhard[1], Cor Semeins[2], Lukas J.A.C. Hawinkels[3,4], Christian Poelma[5], Peter ten Dijke[3], Astrid Bakker[2], Beerend P. Hierck[6] and Dorien J.M. Peters[1]

[1] Department of Human Genetics, Leiden University Medical Center, 2300 RC Leiden, The Netherlands

[2] Department of Oral Cell Biology, Academic Centre for Dentistry Amsterdam (ACTA), University of Amsterdam and VU University Amsterdam, 1081 LA Amsterdam, The Netherlands

[3] Department of Molecular Cell Biology, Cancer Genomics Centre Netherlands, Leiden University Medical Center, 2300 RC Leiden, The Netherlands

[4] Department of Gastroenterology-Hepatology, Leiden University Medical Center, 2300 RC Leiden, The Netherlands

[5] Laboratory for Aero and Hydrodynamics, Delft University of Technology, 2628 CA Delft, The Netherlands

[6] Department of Anatomy and Embryology, Leiden University Medical Center, 2300 RC Leiden, The Netherlands

Corresponding author: Prof. D.J.M. Peters
Department of Human Genetics
Leiden University Medical Center
2300 RC Leiden, The Netherlands
Phone: +31-(0)71-5269490
Fax: +31-(0)71-5268285
E-mail: D.J.M.Peters@lumc.nl

Supplementary figures

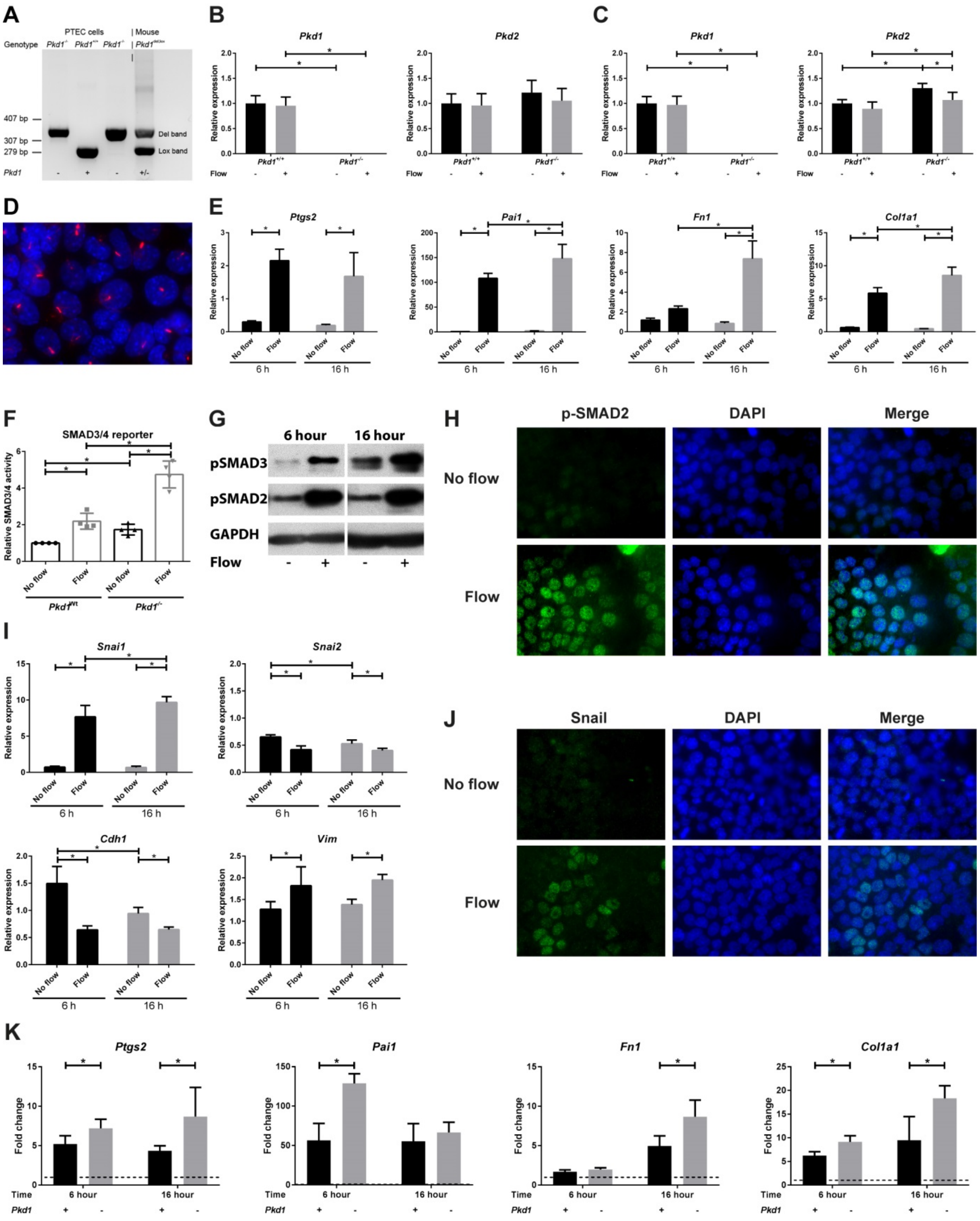

Fig. S1 Activation of SMAD2/3 signaling by fluid-flow in ciliated *Pkd1*$^{-/-}$ PTECs.

a *Pkd1* gene knockout was confirmed by PCR on DNA of *Pkd1*$^{-/-}$ PTECs, which only showed the deletion band, indicating deletion of exon 2-11 of the *Pkd1* gene. *Pkd1*$^{+/+}$ cells only showed the lox band, indicating that the floxed region was still present. As control the *Pkd1*$^{\text{del/lox}}$ mouse showed both

bands. Primers used LoxF: ACCCTTCCCTGAGCCTCCAC; LoxR: CCACAGGGGAAGCCATCATA; DelF: CACTGTGGTGCGGGGTTATC.

b-c *Pkd1* mRNA expression (exon 1-3) is absent in *Pkd1*$^{-/-}$ PTECs, while *Pkd2* expression is not reduced in *Pkd1*$^{-/-}$ PTECs. Expression of *Pkd1* or *Pkd2* was virtually not altered by fluid shear stress, as measured by quantitative PCR. Cone-plate induced fluid-flow in *Pkd1*$^{+/+}$ and *Pkd1*$^{-/-}$ PTECs at t = 6 (**b**) or 16 (**c**) hr; *Hprt* served as housekeeping gene to correct for cDNA input; data normalized to unstimulated PTECs; n=5 per condition; * indicates P < 0.05 using two-way ANOVA.

d Serum starvation induces cilia formation in *Pkd1*$^{-/-}$ PTECs. Cilia are visualized using anti-acetylated α-tubulin antibodies (red) and nuclei are stained with DAPI (blue).

e Relative expression of *Ptgs2* (COX2) and *Pai1* (plasminogen activator inhibitor 1; *Serpine1*), *Fn1*(EDA region; fibronectin) and *Col1a1* (collagen, type I, alpha 1) is increased upon fluid-flow, as measured by quantitative PCR. Cone-plate induced fluid-flow in *Pkd1*$^{-/-}$ PTECs at t = 6 or 16 hr; *Hprt* served as housekeeping gene to correct for cDNA input; data normalized to unstimulated PTECs at 6 hr presented in Fig. 1; n=5 per condition; * indicates P < 0.05 using two-way ANOVA.

f SMAD3-SMAD4 ($GACA_{12}$-Luciferase) transcriptional reporter activity was elevated, as measured upon 20 hr of fluid-flow stimulation. Data normalized to unstimulated PTECs; n=4 per condition; * indicates P < 0.05 using two-way ANOVA.

g Western blot analysis of p-SMAD2 and p-SMAD3 shows increased phosphorylation upon 6 hr and 16 hr fluid-flow stimulation. GAPDH served as loading control.

h Nuclear accumulation of p-SMAD2 (green; t = 6 hr, IF). Nuclei are visualized with DAPI (blue).

i Relative expression of *Snai1* (Snail) and *Vim* (vimentin) is increased, while relative expression of *Snai2* (Slug) and *Cdh1* (E-cadherin) is reduced in *Pkd1*$^{-/-}$ PTECs stimulated with fluid-flow, as measured by quantitative PCR. Cone-plate induced fluid-flow in *Pkd1*$^{-/-}$ PTECs at t = 6 or 16 hr; *Hprt* served as housekeeping gene to correct for cDNA input; data normalized to unstimulated PTECs at 6 hr presented in Fig. 1; n=5 per condition; * indicates P < 0.05 using two-way ANOVA.

j Nuclear accumulation of Snail (green; t = 6 hr, IF). Nuclei are visualized with DAPI (blue).

k Relative expression of *Ptgs2*, *Pai1*, *Fn1* and *Col1a1* shown as fold change induction by fluid shear stress (cone-plate) compared to the no flow control (dashed line), as measured by quantitative PCR. Comparison between *Pkd1*$^{+/+}$ and *Pkd1*$^{-/-}$ PTECs at t = 6 or 16 hr showed stronger induction of target genes by fluid shear stress in *Pkd1*$^{-/-}$ PTECs; *Hprt* served as housekeeping gene to correct for cDNA input; data normalized to unstimulated PTECs (dashed line); n=5 per condition; * indicates P < 0.05 using two-tailed Student's *t*-test

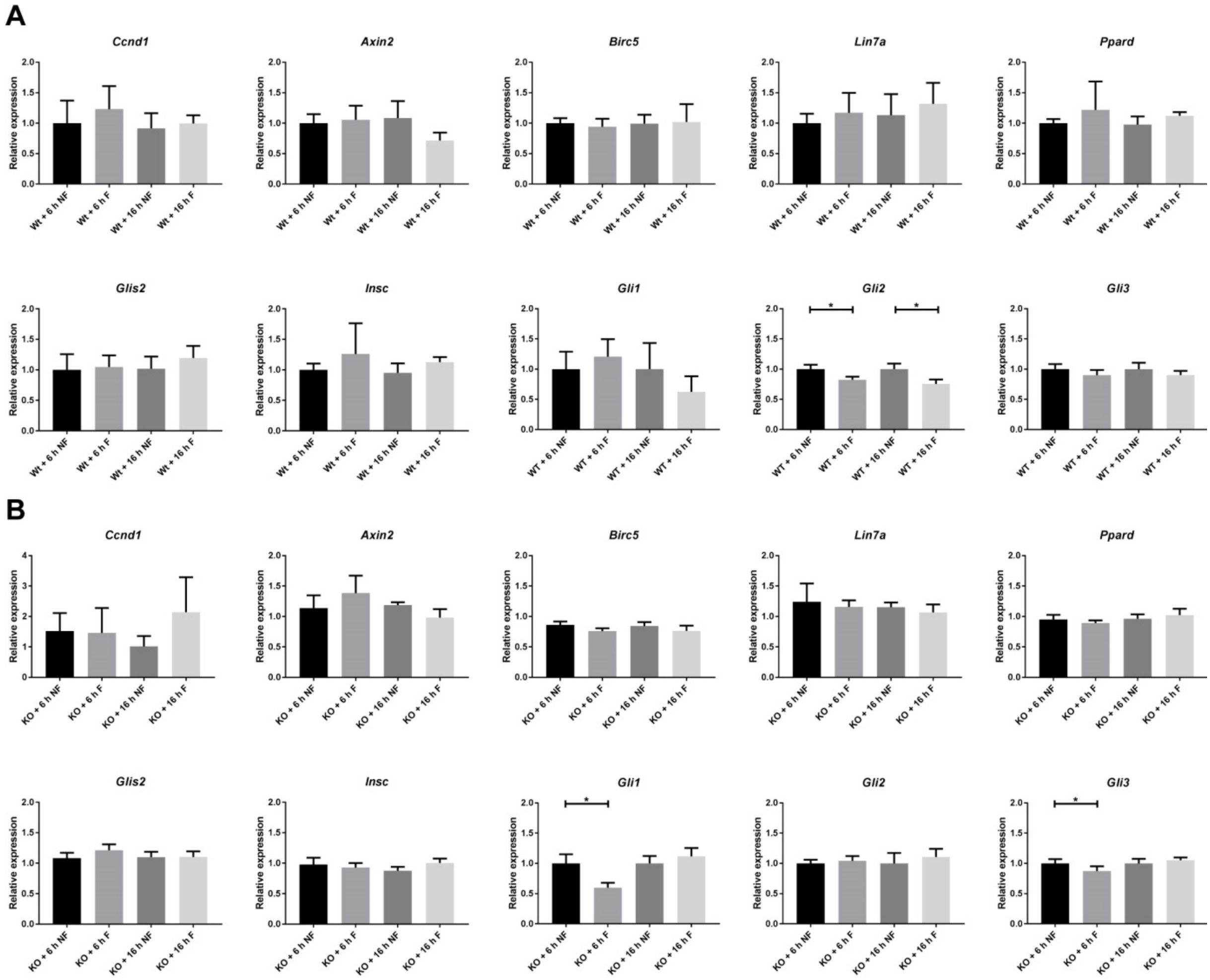

Fig. S2 Expression of Wnt and Hedgehog targets in PTECs upon fluid-flow stimulation.

Relative expression of Wnt target genes (*Ccnd1, Axin2, Birc5, Lin7a, Ppard, Glis2, Insc*) and hedgehog targets (*Gli1, Gli2, Gli3*) is virtually not altered by fluid shear stress. Cone-plate induced fluid-flow in *Pkd1*$^{+/+}$ (**a**) and *Pkd1*$^{-/-}$ (**b**) PTECs at t = 6 or 16 h. Wnt target gene expression was measured by reverse transcriptase multiplex ligation-dependent probe amplification (RT-MLPA) as described previously (Leonhard *et al.* (2008) BMC Biotechnol., 8: 18). Briefly, cDNA was synthesized from total RNA and hybridized to probes (sequences available upon request) in a reaction containing MLPA probe mix and SALSA MLPA buffer (MRC-Holland) by incubation at 95°C for 1 min followed by 60°C for 4 h. Ligation of annealed oligonucleotides was performed at 54°C for 15 min followed by ligase inactivation at 98°C for 5 min. Products were amplified by PCR using SalsaTaq (MRC-Holland) and FAM or HEX-labeled primers. Amplified samples were mixed with Hi-Di formamide containing GeneScan-500 ROX size standard (Applied Biosystems), heated for 5 min at 95°C, and run on a 3730 DNA analyzer (Applied Biosystems). Data was analyzed using GeneScan 3.5 analysis software. Expression of housekeeping genes *Ywhaz* and *Hprt* served as reference for cDNA input. Peak ratios of target genes and housekeeping genes were calculated and results were normalized to unstimulated PTECs. n=3 per condition. Hedgehog targets were measured by quantitative PCR. *Hprt* served as housekeeping gene to correct for cDNA input; data normalized to unstimulated PTECs; n=5 per condition; * indicates $P < 0.05$ using one-way ANOVA

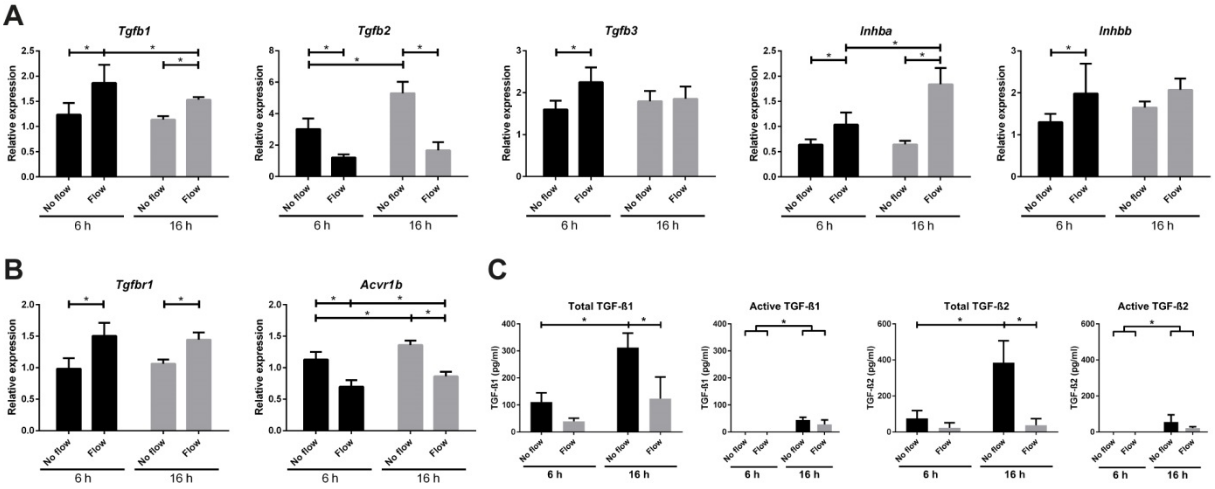

Fig. S3 Expression of ligands and receptors in *Pkd1*$^{-/-}$ PTECs upon fluid-flow stimulation.

a Relative expression of *Tgfb1, Tgfb2, Tgfb3, Inhba, Inhbb* and **b** *Tgfbr1 (Alk5)* and *Acvr1b (Alk4)* mRNA in *Pkd1*$^{-/-}$ PTECs upon fluid-flow. Cone-plate induced fluid-flow at t = 6 or 16 hr; qPCR, *Hprt* served as housekeeping gene to correct for cDNA input; data normalized to unstimulated PTECs at 6 hr presented in Fig. 3; n=5 per condition; * indicates $P < 0.05$ using two-way ANOVA, followed by post-hoc Fisher's LSD multiple comparison.

c Levels of total and active TGF-β1 and TGF-β2 in the medium of *Pkd1*$^{-/-}$ PTECs collected after 6 or 16 hr fluid-flow. TGF-β3 levels in medium and TGF-β1, 2 and 3 levels in cell lysates were below the detection limit. Cone-plate induced fluid shear stress; TGF-β levels measured by ELISA; n=5 per condition; * indicates $P < 0.05$ using two-way ANOVA, followed by post-hoc Fisher's LSD multiple comparison

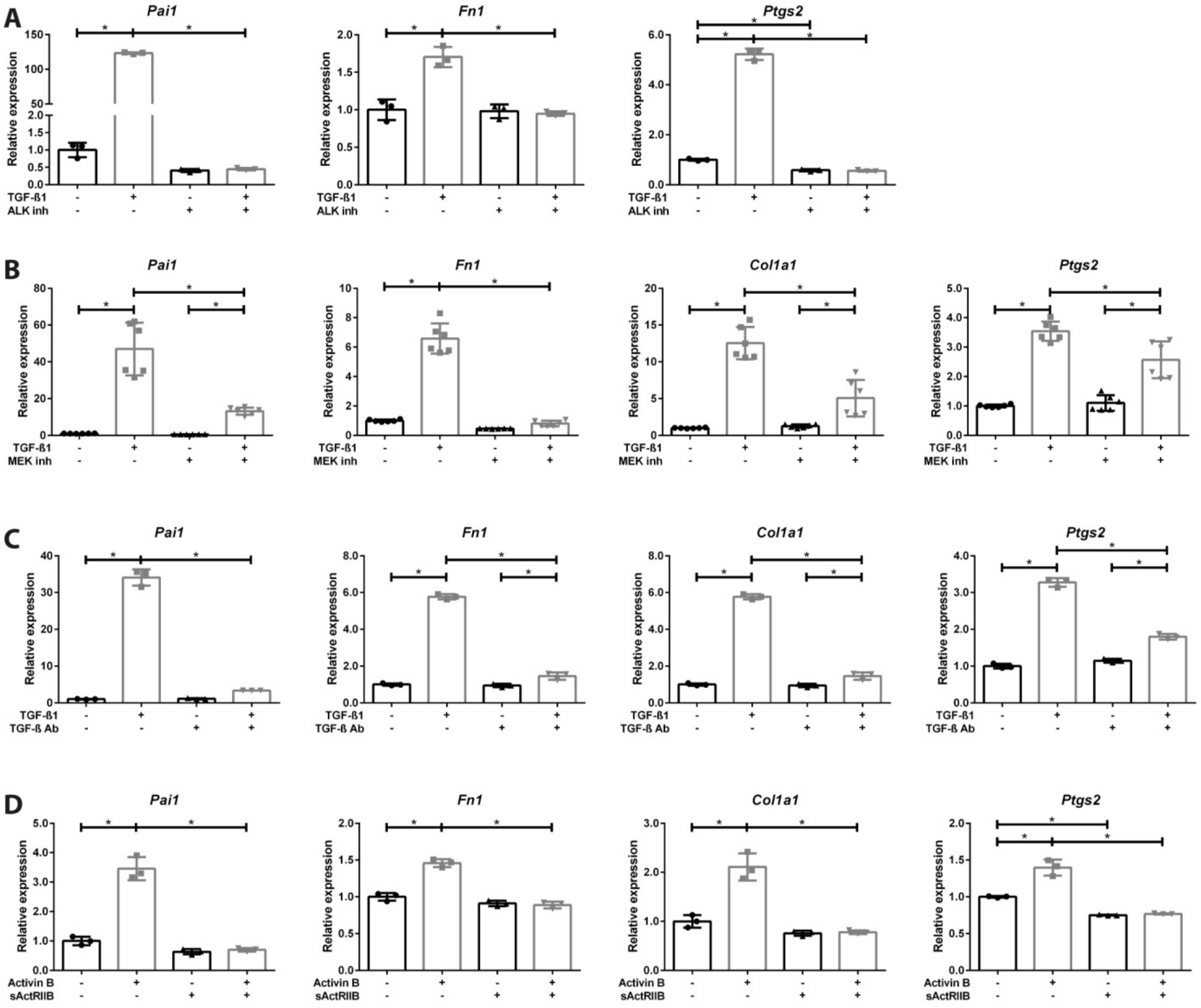

Fig. S4 Expression of SMAD2/3 targets upon TGF-β1 or activin B stimulation when using inhibitors.

a-c TGF-β1 induced expression of SMAD2/3 targets *Pai1, Fn1, Col1a1 and Ptgs2*, was decreased with 10 µM ALK4/5/7 inhibitor (**a**; n=3), 10 µM MEK inhibitor (**b**; n=6) or 10 µg/ml TGF-β neutralizing Ab (**c**; n=3).

d Activin B induced expression of SMAD2/3 targets *Pai1, Fn1, Col1a1 and Ptgs2*, was decreased with 5 µg/ml sActRIIB-Fc (n=3).

Relative mRNA expression measured by qPCR at t = 4 hr (**a**) or 16 hr (**b-d**). *Hprt* served as housekeeping gene to correct for cDNA input; data was normalized to unstimulated controls. * indicates $P < 0.05$ using two-way ANOVA, followed by post-hoc Fisher's LSD multiple comparison. ALK inh = 10 µM ALK4/5/7 inhibitor (LY-364947); MEK inh = 10 µM MEK1/2 inhibitor (Trametinib, GSK1120212). TGF-β Ab = 10 µg/ml TGF-β neutralizing Ab (clone 2G7). sActRIIB = 5 µg/ml soluble activin receptor-IIB fusion protein

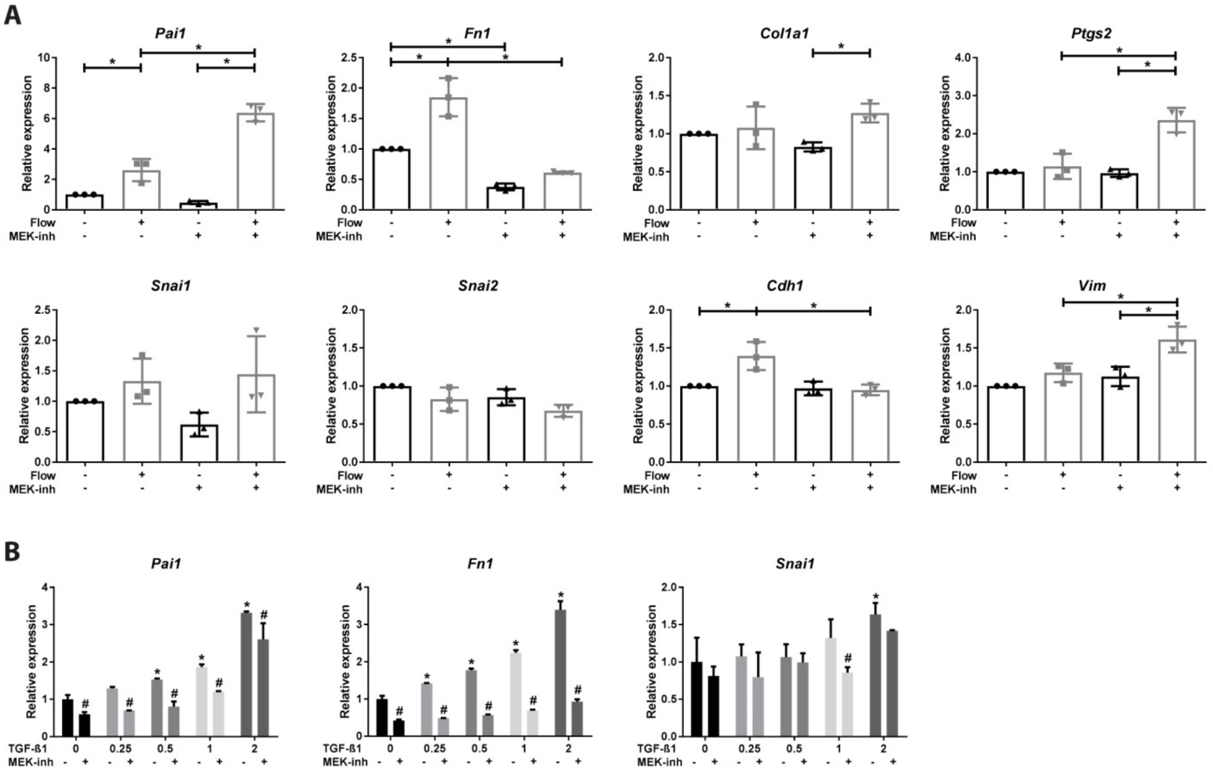

Fig. S5 MEK inhibition modulates fluid-flow or TGF-β1 induced expression of SMAD2/3 target genes.

a MEK inhibition (5 µM Trametinib; GSK1120212) reduces fluid-flow increased expression of *Fn1*, while fluid-flow increased expression of *Pai1*, *Col1a1*, *Ptgs2* and *Snai1* is further elevated. Parallel plate flow-chamber induced fluid-flow in PTECs at t = 16 hr; qPCR, *Hprt* served as housekeeping gene to correct for cDNA input; data normalized to unstimulated controls (fold change); n=3 per condition. * indicates $P < 0.05$ by two-way ANOVA, followed by post-hoc Fisher's LSD multiple comparison.

b *Pai1* and *Fn1* expression was reduced by MEK inhibition (10 µM Trametinib; GSK1120212) upon low dose TGF-β1 (0.25-2 ng/ml) stimulation, as measured by quantitative PCR; *Hprt* served as housekeeping gene to correct for cDNA input; data normalized to unstimulated controls; n=2 per condition. * indicates significant difference compared to unstimulated control (0 ng/ml TGF-β1) or # significant difference upon MEK inhibition (P < 0.05 by two-way ANOVA, followed by post-hoc Fisher's LSD multiple comparison)

Supplementary tables

Table S1 Primer sequences used for qPCR.

Gene	Accession #	Forward primer	Reverse primer
Pai1	NM_008871.2	GCCAACAAGAGCCAATCAC	ACCCTTTCCCAGAGACCAG
Fn1 (EDA)	NM_010233.2	AATCCAGTCCACAGCCATTCC	CCTGTCTTCTCTTTCGGGTTCA
Col1a1	NM_007742.4	TGACTGGAAGAGCGGAGAGT	AGACGGCTGAGTAGGGAACA
Ptgs2	NM_011198.4	ACTGGGCCATGGAGTGGA	ACCTGAGTGTCTTTGACTGTGG
Snai1	NM_011427.3	CTTGTGTCTGCACGACCTG	CAGTGGGAGCAGGAGAATG
Snai2	NM_011415.2	GAACTGGACACACACACAGTTATT	TGCCGACGATGTCCATACAG
Cdh1	NM_009864.3	ATCCTCGCCCTGCTGATT	ACCACCGTTCTCCTCCGTA
Vim	NM_011701.4	CCAACCTTTTCTTCCCTGAA	TGAGTGGGTGTCAACCAGAG
Tgfb1	NM_011577.2	ACTATTGCTTCAGCTCCACAGA	AAGTTGGCATGGTAGCCCTT
Tgfb2	NM_009367.3	CAGGAGTGGCTTCACCACAA	TCAATACCTGCAAATCTCGCCT
Tgfb3	NM_009368.3	AGGATCACCACAACCCACAC	CCAGGTTGCGGAAGCAGTAA
Inhba	NM_008380.1	GACCTCGGAGATCATCACCTT	TGCCTTCCTTGGAAATCTCA
Inhbb	NM_008381.3	CGAGATCATCAGCTTTGCAG	CATAGGGGAGCAGTTTCAGG
Tgfbr1	NM_009370.3	ACATCAGGGTCTGGATCAGGTT	CGACCTTTGCCAATGCTTTCTT
Avcr1b	NM_007395.3	CGAAGATGCAATTCTGGAGGAG	CCGTAGCTTCTGGTCACATACA
Gli1	NM_010296.2	CGACCTGCAAACCGTAATC	AGAGATGGCCGTAGGAACC
Gli2	NM_001081125.1	TGTGCAGTGGAATGAGGTG	TTGCTGTGAGGGAAAGGAG
Gli3	NM_008130.2	GCCATTCACAGTCCAGGTC	TTCCCGCTTTGAGGTAGTG
Pkd1	NM_013630.2	GCCACCGCGCTAGACCTG	TAGCAAACACGCCTTCTTCTAATG
Pkd2	NM_008861.3	GGGAAGCATCTCCAGTGGG	GATGCTGCCAATGGAGTGC
